# A Rare Cause of Paraplegia: Myeloid Sarcoma

**DOI:** 10.4274/tjh.2017.0423

**Published:** 2018-08-05

**Authors:** Esra Arslantaş, Cengiz Bayram, Işık Odaman Al, Ezgi Uysalol, Ayça İribaş, Hilal Akı, İbrahim Adaletli, Ali Ayçiçek, Nihal Özdemir

**Affiliations:** 1 University of Health Sciences, Kanuni Sultan Süleyman Traning and Research Hospital, Clinic of Pediatric Hematology and Oncology, İstanbul, Turkey; 2 İstanbul University Institute of Oncology, Department of Radiation Oncology, İstanbul, Turkey; 3 İstanbul University Cerrahpaşa Faculty of Medicine, Department of Pathology, İstanbul, Turkey; 4 İstanbul University Cerrahpaşa Faculty of Medicine, Department of Radiology, İstanbul, Turkey

**Keywords:** Myeloid sarcoma, Children, Paraplegia

## To the Editor,

Myeloid sarcoma (MS), also known as granulocytic sarcoma or chloroma, is a rare extramedullary tumor consisting of myeloblasts or immature myeloid cells that disrupt the normal architecture of the involved tissue and typically occurs concurrently with acute myeloid leukemia (AML) [1,2]. It can also occur in association with accelerated-phase chronic myeloid leukemia or myelodysplastic syndrome; as an extramedullary relapse of AML, including in the post-bone marrow transplant setting; and occasionally as the first presenting manifestation, even before bone marrow involvement [3,4]. Bone, periosteum, skin, orbit, lymph nodes, the gastrointestinal tract, and the central nervous system are the most commonly involved sites in patients presenting with MS; however, skin and orbital localizations are the most often reported sites in children [4]. Here we present a 4-year-old male patient who was referred to the pediatric hematology oncology clinic due to a thoracolumbar mass and subsequently diagnosed with MS.

**Figure 1:**
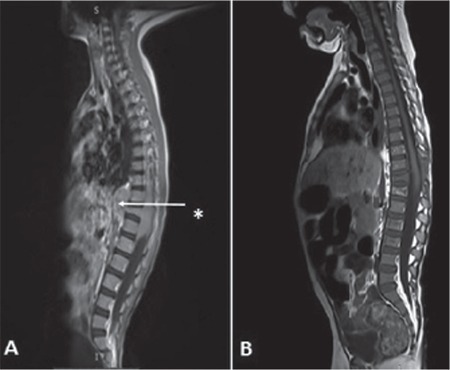
A) Sagittal T1-weighted MRI image showing an epidural, hypointense, craniocaudal mass of 4.5 cm in diameter compressing the spinal cord at the level of D 10-12; B) image of the mass 1 month before completion of acute myeloid leukemia maintenance therapy

A 4-year-old boy was referred to the pediatric hematology oncology clinic with the complaint of hemiparesis and a subsequent thoracolumbar mass was detected by magnetic resonance imaging (MRI) (Figure 1A). On physical examination, bilateral lower extremity paralysis was noted and deep tendon reflexes were absent. Complete blood count and blood biochemical analysis were normal, and no blasts were detected on peripheral blood film. Bone marrow aspiration showed 30% blasts compatible with AML. The pathology of the mass revealed MS. After administration of radiotherapy, given at a dose of 18 Gy in 10 daily fractions in 2 weeks, and dexamethasone therapy, the patient achieved neurological improvement. He was treated with the AML-Berlin Frankfurt Münster 2012 protocol and achieved both remission and mass reduction following AML induction chemotherapy. The patient is still in remission without any residual tumor on follow-up MRI (Figure 1B).

MS may occur at any site of the body, and therefore clinical manifestations of MS exhibit diversity depending on the specific location and size, which leads to significant diagnostic challenges, in particular in patients without initial bone marrow involvement. Incorrect diagnosis of malignant lymphoproliferative disorders, Ewing’s sarcoma, thymoma, melanoma, round blue cell tumors, or poorly differentiated carcinoma has been reported at a rate of 25%-47% in patients subsequently diagnosed with MS. In this regard, any atypical cellular infiltrate should raise the suspicion of MS to make a correct diagnosis in a timely manner and to allow for proper management [2,4,5]. Diagnostic tools for the correct diagnosis of MS are also important in this context and should include MRI and/or computed tomography scan for evaluation of the size and location of the tumor and for distinguishing the tumor from other lesions, morphological and flow cytometric analysis of bone marrow and peripheral blood, or biopsy of the tumor and immunohistochemical staining in patients without bone marrow involvement [4]. Treatment of MS includes AML-based protocols and, as in our case, surgery and/or radiotherapy may be indicated for symptomatic lesions or tumors causing local organ dysfunction [5]. Considering the most common presentation sites in children with MS, which are skin and orbital localizations, the current patient is presented to highlight a rarely encountered presenting feature of MS.
